# B cell response after SARS-CoV-2 mRNA vaccination in people living with HIV

**DOI:** 10.1038/s43856-023-00245-5

**Published:** 2023-01-30

**Authors:** Jacopo Polvere, Massimiliano Fabbiani, Gabiria Pastore, Ilaria Rancan, Barbara Rossetti, Miriam Durante, Sara Zirpoli, Enrico Morelli, Elena Pettini, Simone Lucchesi, Fabio Fiorino, Mario Tumbarello, Annalisa Ciabattini, Francesca Montagnani, Donata Medaglini

**Affiliations:** 1 Laboratory of Molecular Microbiology and Biotechnology, Department of Medical Biotechnologies, University of Siena, Siena, Italy; 2 Department of Medical Sciences, Infectious and Tropical Diseases Unit, University Hospital of Siena, Siena, Italy; 3 Department of Medical Biotechnologies, University of Siena, Siena, Italy

**Keywords:** Adaptive immunity, Vaccines, HIV infections, B cells, Viral infection

## Abstract

**Background:**

Limited longitudinal data are available on immune response to mRNA SARS-CoV-2 vaccination in people living with HIV (PLWHIV); therefore, new evidence on induction and persistence of spike-specific antibodies and B cells is needed.

**Methods:**

In this pilot study we investigated the spike-specific humoral and B cell responses up to six months after vaccination with two doses of mRNA vaccines in 84 PLWHIV under antiretroviral therapy compared to 79 healthy controls (HCs).

**Results:**

Spike-specific IgG persisted six months in PLWHIV with no significant differences compared to HCs, even though a significantly lower IgG response was observed in patients with CD4^+^ T cells < 350/mmc. The frequency of subjects with antibodies capable of inhibiting ACE2/RBD binding was comparable between PLWHIV and HCs a month after the second vaccine dose, then a higher drop was observed in PLWHIV. A comparable percentage of spike-specific memory B cells was observed at month six in PLWHIV and HCs. However, PLWHIV showed a higher frequency of spike-specific IgD^-^ CD27^-^ double-negative memory B cells and a significantly lower rate of IgD^-^ CD27^+^ Ig-switched memory B cells compared to HCs, suggesting a reduced functionality of the antigen-specific memory B population.

**Conclusions:**

The mRNA vaccination against SARS-CoV-2 elicits humoral and B cell responses quantitatively similar between PLWHIV and HCs, but there are important differences in terms of antibody functionality and phenotypes of memory B cells, reinforcing the notion that tailored vaccination policies should be considered for these patients.

## Plain language summary

SARS-CoV-2 vaccination has been demonstrated to protect people from severe COVID-19 and death. This is achieved through the induction of a specific immune response that recognizes and responds to the virus. Limited data are available on the immune response to SARS-CoV-2 vaccination in people living with HIV (PLWHIV). In this study, we evaluated the immune response up to six months after vaccination with two doses of vaccines in PLWHIV being treated with the standard antiretroviral therapy. We show that the immune response observed in PLWHIV is broadly similar to that in healthy subjects but that there are some differences in the cells induced as part of the immune response. We therefore suggest that specific vaccination policies should be considered for these PLWHIV.

## Abstract (web summary)

Polvere et al. investigate B cell responses after SARS-CoV-2 vaccination in people living with HIV undergoing anti-retroviral therapy. Spike-specific humoral and B cell responses persist for at least six months although a reduced ACE2/RBD binding inhibition capacity and different memory B phenotypes are observed compared to healthy controls.

## Introduction

People affected by immunodeficiency conditions, such as HIV infection, are known to be more susceptible to infections but the effect of impaired immunity in SARS-CoV-2 infection and COVID-19 progression is still hard to be stated^1^. There is no consensus on the role of chronic HIV infection in COVID-19 outcome, since in some cohorts it appears as a negligible risk factor but other studies highlight the higher rate of hospitalization and death in people living with HIV (PLWHIV), especially for patients with low CD4^+^ T cells count and with detectable viremia^2–4^. Moreover, it was demonstrated that SARS-CoV-2 infected PLWHIV under effective antiretroviral regimens (ART), with a good immunological recovery and adequate virological control, can develop humoral and T cell immune responses similar to healthy controls (HCs)^5^. CD4^+^ T cells/mmc count and the CD4^+^/CD8^+^ T cell ratio, as well the ART and compliance, have also been shown to be relevant factors to mount an effective immune response to influenza or pneumococcus vaccines^6,7^.

Differently from what observed in other immunocompromised subjects, such as patients with multiple comorbidities and elderly people^8–11^, most PLWHIV present high immune responsiveness for SARS-CoV-2 mRNA vaccines^12,13^, yet the anti-spike immunity can even be absent in PLWHIV with profound immune dysfunction vaccinated with two doses^14^.

As a consequence of waning immune protection against SARS-CoV-2 infection and the emerging viral variants of concerns, the FDA have recently approved a fourth dose with mRNA vaccines for immunocompromised^15^. Whether or when to globally plan future booster doses in PLWHIV, is a burning question. Immunological studies on healthy subjects vaccinated with nanoparticles-based mRNA formulations^16^ clearly show the persistence of immune memory cells and circulating spike-specific antibodies at least up to 6 months from vaccination, despite a physiological decline in the first 2 months after the second dose^17–19^. Up to date, longitudinal data regarding SARS-CoV-2 vaccine humoral response in PLWHIV do not suggest a reduced persistence of circulating antibody compared to healthy subjects^20–22^. However, there are no studies that investigate in PLWHIV the spike-specific memory B cells upon SARS-CoV-2 vaccination. This aspect is particularly relevant also considering that there are indications that the B-cell compartment of PLWHIV presents phenotypic and functional alterations with increased rate of CD27^- ^IgD^-^ double-negative (DN) cells as well as atypical or tissue-like memory cells (CD21^- ^CD27^-^) ^23–25^. Therefore, it is of primary importance to investigate the induction and persistence of spike-specific memory B cells, essential to guarantee long-term immunity and reactivation at the antigen encounter, in PLWHIV upon SARS-CoV-2 vaccination.

In this pilot study, we profiled the spike-specific B-cell response and the kinetic of the antibody response against SARS-CoV-2 for 6 months after the mRNA vaccination in PLWHIV, that are currently on ART, and we compared data with healthy controls. We observed comparable spike-specific antibody titers but reduced ACE2/RBD inhibition activity and different phenotypes of memory B cells.

## Methods

### Study cohort

Plasma and peripheral blood mononuclear cells (PBMCs) were obtained from adult PLWHIV under different ART regimens and from adult healthy controls (HCs) who received two doses of mRNA vaccines, either mRNA-1273 (Moderna) or BNT162b2 (Pfizer), 3 or 4 weeks apart according to national schedules. A total of 84 PLWHIV were enrolled in the context of the PATOVAC study, while 79 healthy subjects were enrolled in the IMMUNO_COV study. All participants were recruited at the Infectious and Tropical Diseases Unit, Azienda Ospedaliera Universitaria Senese (Siena, Italy), where they provided written informed consent before participation to the study. Inclusion criteria were age ≥ 18 years and adhesion to the COVID-19 vaccination campaign (for both studies), confirmed diagnosis of HIV infection (only for PATOVAC study). Exclusion criteria were pregnancy, withdrawal of consent or refusal to participate (for both studies), administration of high-dose steroids or other immunosuppressant drugs, comorbidities associated with relevant immunosuppression (e.g., active cancer, organ transplantation) other than HIV itself, clinical problems for collecting additional blood samples beyond the amount required for routine care and participation to other clinical trials (for PATOVAC study), being affected by any immunocompromising condition (congenital, acquired, or drug-related; for IMMUNO_COV study). The studies were performed in compliance with all relevant ethical regulations and the protocol was approved by local Ethical Committee for Clinical experimentation of Regione Toscana Area Vasta Sud Est (CEAVSE; protocol code 19479 PATOVAC v1.0 of 03 Mar 2021, approved on 15 Mar 2021 and protocol code 18869 IMMUNO_COV v1.0 of 18 Nov 2020, approved on 21 Dec 2020 for HCs). Clinical data collection and management were carried out using the software REDCap (Research Electronic Data Capture, Vanderbilt University, TN, USA)^26^.

### Plasma and peripheral blood mononuclear cells isolation

Venous blood samples were collected in heparin-coated blood tubes (BD Vacutainer) at baseline (pre v1), before the second dose (pre v2), and 1, 2 and 5 months post the second dose (+30 v2, +60 v2, and +150 v2, respectively). PBMCs were isolated by density-gradient sedimentation, using Ficoll-Paque (Lymphoprep, Meda, Italy). Isolated PBMCs were then cryopreserved in a cell recovery medium [10% DMSO (Thermo Fisher Scientific) and 90% heat-inactivated fetal bovine serum (Sigma Aldrich)] and stored in liquid nitrogen until used. Plasma samples were stored at −80 °C.

### Enzyme-linked immunosorbent assay (ELISA)

 Spike-specific IgG were tested on Maxisorp microtiter plates (Nunc, Denmark) coated with recombinant SARS-CoV-2 spike S1 + S2 ECD (1 μg/mL protein; Sino Biological), as previously reported^10^. Briefly, plates were blocked (PBS and 5% skimmed milk powder, 0.05% Tween 20),  and added with heat-inactivated plasma samples titrated in twofold dilutions, in duplicate. Anti-human horseradish peroxidase (HRP)-conjugated IgG was added in diluent buffer for 1 h at RT, and then plates were developed with 3,3’,5,5’-Tetramethylbenzidine (TMB; Thermo Fisher Scientific) substrate for 10 min at RT, followed by addition of 1 M stop solution. Absorbance at 450 nm was measured on Multiskan FC Microplate Photometer (Thermo Fisher Scientific).

### ACE2/RBD inhibition assay

ACE2/RBD binding inhibition was tested with a SARS-CoV-2 surrogate virus neutralization test (sVNT) kit (cPass™, Genscript), according to the manufacturer protocol and as previously reported^17^. Briefly, plasma samples, positive and negative controls were diluted 1:10 in dilution buffer, mixed 1:1 with HRP-RBD buffer and incubated for 30 min at 37 °C. 100 µl/well of each mixture were added to ACE2-coated 96 wells plates and incubated for 15 min at 37 °C. Plates were washed four times and tapped dry. 100 µl of TMB solution were added to each well and plates were developed for 15 min at RT (20–25°). After that, the reaction was quenched adding 50 µl of the stop solution to each well and the OD at 450 nm was immediately read with Multiskan FC Microplate Photometer (Thermo Fisher Scientific). Results are reported as follows: percentage inhibition = (1 – sample OD value/negative control OD value) * 100. Inhibition values ≥ 30% are regarded as positive results, as indicated by the manufacturer.

### Multiparametric flow cytometry

Two million of PBMCs were incubated with BD human FC block (BD Biosciences) and then stained with recombinant biotinylated spike S1 + S2 ECD-His (Sino Biological) conjugated with SA-R-Phycoerythrin (PE) and recombinant biotinylated-receptor binding domain  (RBD; BioLegend) conjugated with SA-Allophycocyanin (APC), together with the following fluorescent antibodies (clone; dilution used): CD3-BV650 (clone OKT3; 1:200); CD21-FITC (clone B-LY4; 1:50), CD19-BUV395 (clone SJ25C1; 1:100), CD10-PECF594 (clone HI10A; 1:200), IgM-BV605 (clone G20-127; 1:50), IgD-BV711 (clone IA6-2; 1:100), CD27-BV786 (clone O323; 1:50), CD11c-BB700 (clone 3.9; 1:50), CD20-APCH7 (clone 2H7; 1:100), CD38-BUV737 (clone HB7; 1:400), IgG-PE-Cy7 (clone G18-145; 1:100; all from Becton Dickinson), IgA-Vio blue (clone IS11-8E10; 1:100; Miltenyi Biotec). Following surface staining, cells were washed once with PBS and labeled with Zombie Aqua Fixable Viability Kit (Thermofisher) according to the manufacturer instruction. Cells were fixed in BD fixation solution (BD Biosciences) and acquired with SO LSRFortessa X20 flow cytometer (BD Biosciences). Data analysis was performed using FlowJo v10 (TreeStar, USA).

### t-SNE visualization

The B cell population analyzed in our dataset was gated as live, singlet, CD3^−^ CD19^+^ cells using FlowJo v10 (TreeStar, USA) while antigen-specific B cells were gated as S^+^ RBD^+^ cells. B cell files were then exported as.fcs file and imported in R environment as flowSet object, that was then compensated with FlowCore package 2.6.0 and logicle transformed^27^. t-Distributed Stochastic Neighbor Embedding (t-SNE) dimensionality reduction^28^ was performed with Rtsne package v0.15. Expression values of each marker were normalized as z-scores (mean = 0 and standard deviation = 1). An equal number of cells (25000) were downsampled from PLWHIV and healthy donor samples, then Rtsne function was run setting perplexity = 100, selected as optimal parameter value in a range between 5 and 200. B cells were analyzed with manual gating and labels of different B-cell populations were imported in R environment using GetFlowJoLabels function from FlowSOM package (v2.2.0) and visualized with function Contour from FlowViz package (v1.58.0).

### Statistics and reproducibility

Descriptive statistics [number, proportion, median, interquartile range (IQR), 95% confidence intervals (CI)] were used to describe the baseline characteristics of patients. Categorical variables were compared between groups using the Chi-square test or Fisher’s exact test, as appropriate. Continuous variables were compared using the non-parametric Mann–Whitney test. For ELISA titers, Mann–Whitney test was used to assess the statistical differences between two groups at each time point, while Kruskal–Wallis test, followed by Dunn’s post-test for multiple comparisons, was used to assess the statistical differences between multiple groups and time points. Mann–Whitney test was used to assess differences in B-cell subpopulations between the two cohort of the study. Fisher’s exact test was used to assess the statistical differences in percentages of ACE2/RBD inhibition positive subjects between groups. Spearman correlation test was used for assessing correlation between absolute CD4^+^ T cells/mmc and log-transformed ELISA titers. Clinical and laboratory variables of PLWHIV potentially associated with log-transformed ELISA titers or ACE2/RBD inhibition percentage were investigated by a linear regression model, while those associated with ACE2/RBD binding inhibition value ≥ 30% were investigated by a logistic regression model; in both models, factors associated with the dependent variables at univariate analysis, together with absolute CD4^+^ T cells count, CD4^+^ T cells percentage and CD4^+^/CD8^+^ ratio, were then evaluated in a multivariate analysis. All statistical analyses were conducted on measurements taken from biologically distinct samples. Sample sizes at each time points were reported in figure legends. Analyses were performed using GraphPad Prism v9 (GraphPad Software, San Diego, CA, USA) and SPSS Software, version 23.0 (SPSS Inc., Chicago, IL).

### Reporting summary

Further information on research design is available in the Nature Portfolio Reporting Summary linked to this article.

## Results

A total of 84 PLWHIV with a median age of 52 years (IQR 46–58) were enrolled in the study, of whom 64 (76.2%) were male and 20 (23.8%) were female. Among them, 57 (67.9%) had > 500 CD4^+^ T cells/mmc (High CD4^+^), 13 (15.5%) were in the 350–500 CD4^+^ T cells/mmc range (Average CD4^+^) and 14 (16.7%) had values < 350 CD4^+^ T cells/mmc (Low CD4^+^). At baseline, 28 (33.3%) PLWHIV showed optimal immunological recovery, defined as CD4^+^ T cells count ≥ 500 cell/mmc plus CD4^+^ T cells % ≥ 30% plus CD4/CD8 ratio ≥ 1^29,30^. Clinical and demographic data of PLWHIV cohorts were collected before the first dose for the statistical analysis and reported in Supplementary Table 1. No changes in treatment or significant variation in CD4^+^ T cells count were observed during the study. Of the 8 patients with baseline HIV-RNA > 50 copies/mL: 3 had an isolated blip, 1 had frequent blips during follow-up, 3 had virological failure with persistent low-level viremia, and 1 had virological failure (but reached virological suppression after 3 months with antiretroviral therapy optimization). In this subgroup, median CD4 count was 603 cells/mmc (IQR 425–783), with 2 (2.4% on total) subjects showing CD4 < 350 cells/mmc. No significant differences in immune responses to the vaccine were observed between patients with detectable vs. undetectable viral load during follow-up (data not shown).

The HCs group was enrolled to match the age and numerosity of PLWHIV. It was composed of 79 healthy volunteers with a median age of 52 years (IQR 45–60) of whom 22 (27.8%) were males and 57 (72.2%) were females. A slightly higher body mass index (BMI) (25.1 vs. 23.7, *P* = 0.037) was observed in PLWHIV. Moreover, HCs were more frequently vaccinated with BNT162b2 vaccine when compared to PLWHIV who mostly received mRNA-1273 (87.3% vs. 48.8%, *P* < 0.001.).

### Spike-specific antibody response following SARS-CoV-2 mRNA vaccination in PLWHIV

**Fig. 1:**
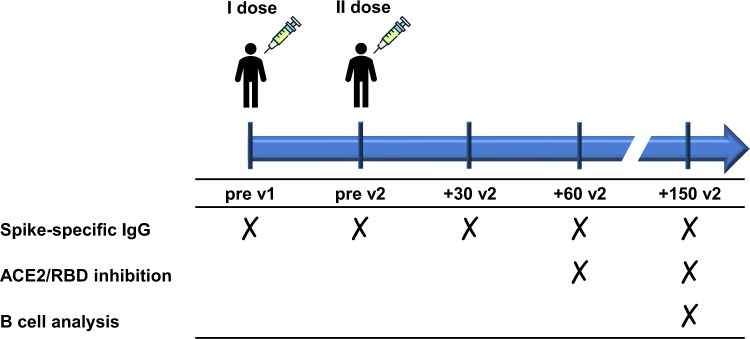
Schematic representation of the study design. PLWHIV (84 subjects) and healthy controls (HCs, 79 subjects) vaccinated with two doses of a SARS-CoV-2 mRNA vaccine (BNT162b2 Pfizer-BioNTech or mRNA-1273 Moderna) 3–4 weeks apart, respectively, were enrolled in the study. Blood samples were collected at pre v1 (day 0, baseline), pre v2, +30 v2 (1 month post second dose), +60 v2 (2 months post second dose), and +150 v2 (5 months post second dose). Plasma samples were tested for spike-specific IgG and ACE2/RBD binding inhibition while PBMCs were analyzed for spike-specific B-cell response.

**Fig. 2:**
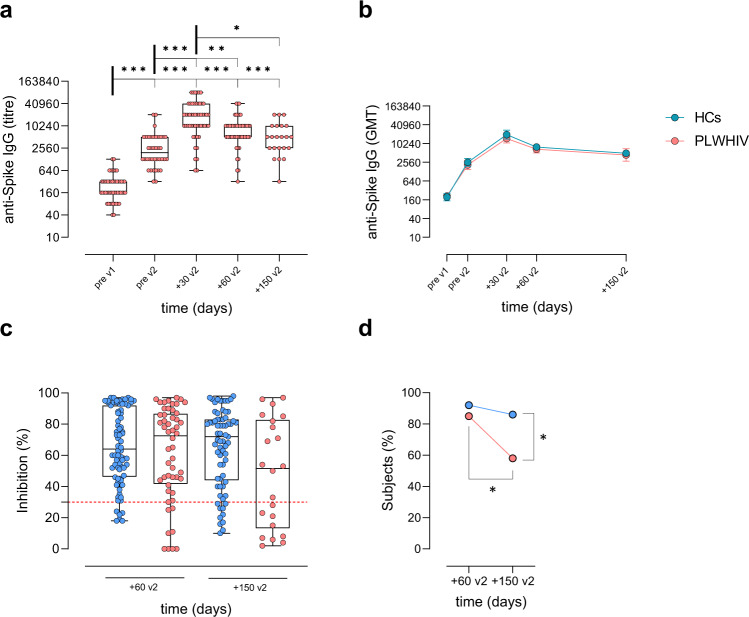
Spike-specific antibody response following SARS-CoV-2 mRNA vaccination. **a** Anti-spike IgG detected at pre v1, pre v2, +30 v2, +60 v2, and +150 v2 in plasma of PLWHIV. Data are presented as box and whiskers diagram showing the minimum and maximum of all the data. Kruskal–Wallis test, followed by Dunn’s post test for multiple comparisons, was used for assessing statistical differences between groups. *P ≤ 0.05; **P ≤ 0.01; ***P ≤ 0.001. **b** Time course of spike-specific IgG response in PLWHIV and HC. Antibody titers are expressed as the reciprocal of the dilution of sample reporting a double OD value compared to the background. Data are presented as geometric mean titers (GMT) with 95% CI. Mann–Whitney test was used for assessing statistical differences between groups at each time point. **c**, **d** Surrogate virus neutralization assay (sVN) performed at +60 v2 and +150 v2. In c, data are reported as ACE2/RBD binding inhibition percentage with box and whiskers diagram showing the minimum and maximum of all the data. A threshold (dotted red line) was placed at 30% inhibition percentage to discriminate between positive and negative samples. Kruskal–Wallis test, followed by Dunn’s post-test for multiple comparisons, was used for assessing statistical differences between groups. The percentage of positive subjects over all samples, tested at each time point, is reported in d. Fisher’s Exact Test was used to assessing statistical differences between groups. *P ≤ 0.05; **P ≤ 0.01. Sample size PLWHIV: pre v1 (*n* = 54), pre v2 (*n* = 38), +30 v2 (*n* = 53), +60 v2 (*n* = 51), +150 v2 (*n* = 22). Sample size HCs: pre v1 (*n* = 25), pre v2 (*n* = 50), +30 v2 (*n* = 52), +60 v2 (*n* = 75), +150 v2 (*n* = 69). All samples were biologically independent.

Spike-specific IgG induced by SARS-CoV-2 vaccination were assessed by ELISA on plasma samples collected from PLWHIV at different time points (Fig. 1). According to Kruskal–Wallis test, anti-spike antibody titers significantly changed throughout the study (*P* ≤ 0.001, Cohen’s *d* 2.857). Anti-spike IgG significantly increased after the first dose, reaching a geometric mean titer (GMT) of 2105 at pre v2 [95% CI 1514 to 2928; titers range 320–20480; *P* ≤ 0.001 vs. pre v1; Fig. 2a]. Antibody titers increased again after the second dose, reaching a peak at +30 v2, with a GMT of 14482 (95% CI 10531 to 19915; titers range 640–81920; *P* ≤ 0.001 vs. pre v1 and pre v2). Significant antibody titers were still observed 2 months post boost (+60 v2) (GMT 6684; 95% CI 5086 to 8785; titers range 320–40960; *P* ≤ 0.001 vs. pre v1 and *P* ≤ 0.01 vs. pre v2) and 5 months post boost (+150 v2) (GMT 4974; 95% CI 3218 to 7690; titers range 320–20480; *P* ≤ 0.001 vs. pre v1) despite a progressive physiological decline over time. No statistically significant differences were observed between 2 months (+60 v2) and 5 months (+150 v2) post-boost (*P* > 0.99), as well as between GMT at pre-boost (pre v2) and at 5 months post-boost (+150 v2) (*P* = 0.46).

The curve of spike-specific IgG observed in PLWHIV perfectly overlaid to the one measured in HCs (Fig. 2b) Both cohorts reached their maximum titers after the second dose (HCs GMT at +7 v2: 19677; 95% CI 14183 to 27300) and their response progressively declined over time. No statistically significant differences were observed between the two groups for any time point of the study.

A stratification based on CD4 + T-cell/mmc (≤ 350 Low CD4^+^, 350–500 Average CD4^+^ and ≥ 500 High CD4^+^) was done to highlight a possible predictive value for the humoral response (Supplementary Fig. 1). The statistical analysis revealed a significant difference in antibody response at +30 v2 between High CD4^+^ (GMT 19038; 95% CI 14328 to 25297) and Low CD4^+^ (GMT 1612; 95% CI 539 to 4821) (*P* ≤ 0.001, Cohen’s d 1.27) and between Average CD4^+^ (GMT 22119; 95% CI 11248 to 43499) and Low CD4^+^ (*P* ≤ 0.001, Cohen’s *d* 2.344; Supplementary Fig. 1). Spearman test was used to assess the correlation between absolute CD4^+^ T cells/mmc and log-transformed ELISA IgG titers. A significant positive correlation was found at pre v2 (*r* = 0.336; *P* ≤ 0.05), +30 v2 (*r* = 0.328; *P* ≤ 0.05) but not at +60 v2 (*r* = 0.148; *P* = 0.31) and +150 v2 (*r* = −0.0248; *P* = 0.90).

We also investigated HIV-related variables associated with IgG titers at +30 v2 by linear regression analysis (Table 1). At multivariate analysis, patients with < 350 cells/mmc confirmed to develop lower IgG titers (mean change −1.02, 95% CI −1.39 to −0.65, *P* ≤ 0.001 when compared to those with CD4^+^ > 350 cells/mmc) after adjusting for having CD4^+^ T cells % ≥ 30% and CD4^+^/CD8^+^ ratio ≥ 1.

**Table 1:** Variables associated with log-transformed IgG titers 1 month post boost dose (+30 v2) (univariate and multivariate linear regression analysis).

	Univariate analysis	*P*	Multivariate analysis	*P*
	Mean change (95% CI)		Adjusted mean change (95% CI)	
Age, per +10 years	0.11 (–0.05/0.26)	0.169		
Male gender	–0.10 (–0.43/0.23)	0.563		
Type of vaccine (mRNA-1273 vs. BNT162b2)	–0.07 (–0.37/0.22)	0.623		
BMI, kg/m^2^	–0.00 (–0.03/0.02)	0.836		
IDU	0.03 (–0.36/0.43)	0.864		
Years from HIV infection, per +10 years	0.03 (–0.12/0.17)	0.731		
CDC stage C	–0.15 (–0.53/0.23)	0.426		
HBV or HCV coinfection	–0.05 (–0.36/0.25)	0.736		
Zenith HIV-RNA, per +1 log copies/mL	–0.01 (–0.28/0.27)	0.946		
CD4 cell count at nadir, per +100 cell/mmc	0.07 (–0.02/0.16)	0.124		
Years from first ART, per +10 years	0.06 (–0.12/0.23)	0.526		
InSTI + 2NRTI	–0.10 (–0.40/0.20)	0.503		
Baseline HIV-RNA <50 cp/mL	0.25 (–0.28/0.79)	0.347		
Time from last HIV-RNA > 50 copies/mL, per +10 years	–0.02 (–0.47/0.44)	0.942		
CD4 cell count at baseline, per +100 cell/mmc	0.04 (–0.01/0.08)	0.102		
CD4 cell count at baseline ≤350 cell/mmc	–1.08 (–1.41/–0.75)	**<0.001**	–1.02 (–1.39/–0.65)	**<0.001**
CD4% ≥ 30%	0.36 (0.09/0.64)	**0.009**	0.01 (–0.30/0.32)	0.947
CD4/CD8 ratio ≥1	0.33 (0.06/0.60)	**0.019**	0.11 /–0.19/0.40)	0.459
Comorbidities:				
Diabetes	–0.42 (–0.94/0.11)	0.120		
Hypertension	0.21 (–0.14/0.59)	0.264		
Chronic lung disease	0.27 (–0.17/0.72)	0.223		
Previous cancer	0.05 (–0.40/0.50)	0.835		

Bold values indicate statistical significance *p* ≤ 0.05.

*ART* antiretroviral therapy, *BMI* body mass index, *CI* confidence intervals, *HBV* hepatitis B virus, *HCV* hepatitis C virus, *IDU* injecting drug users, *InSTI* integrase strand transfer inhibitors, *MSM* men who have sex with men, *NRTI* nucleoside reverse transcriptase inhibitors, *NNRTI* non-nucleoside reverse transcriptase inhibitors, *OIR* optimal immunological recovery, *PI* protease inhibitors.

A surrogate virus neutralization assay was employed to evaluate overtime  the ability of vaccine-induced antibodies to block the ACE2/RBD interaction at +60 v2 and +150 v2. Results were reported as percentages of ACE2/RBD binding inhibition assessed in plasma of each subject (Fig. 2c) and as percentage of patients with positive inhibition values (≥ 30%; Fig. 2d). Two months after the second dose the majority of PLWHIV presented positive inhibition values, without significant differences compared to HCs (84.6% vs. 92.1%; *P* = 0.250, Odds ratio 2.121, 95% CI 0.6486 to 6.731; Fig. 2d). However, the PLWHIV cohort registered a significant reduction in the percentage of patients with a positive value of inhibiting antibodies overtime (84.6% at +60 v2 vs. 58.4% at +150 v2; *P* ≤ 0.05, Odds ratio 3.808, 95% CI 1.156 to 10.88), while the HCs group maintained a stable value (92.1% at +60 v2 vs. 86.2% at +150 v2; *P* = 0.289, Odds ratio 0.5057, 95% CI 0.1718 to 1.375). The frequency of inhibition positive PLWHIV at 5 months post-second dose was significantly lower compared to HCs (58.4% vs. 86.2%, respectively; *P* ≤ 0.05, Odds ratio 4.085, 95% CI 1.349 to 12.33).

We also investigated HIV-related variables associated with ACE2/RBD binding  inhibition values at 2 months post second dose (+60 v2) by linear regression analysis (Supplementary Table 2). A trend toward an association between CD4^+^ T-cell count ≤ 350 cell/mmc at baseline and a lower binding inhibition value was observed at univariate analysis (mean change −19.30%, 95% CI −40.99/2.40, *P* = 0.080), but this was not confirmed after adjusting for having CD4^+^ % ≥ 30% and CD4^+^/CD8^+^ ratio ≥ 1.

Moreover, we explored variables associated with the percentage of inhibition positive PLWHIV at 2 months post boost dose (+60 v2) by logistic regression analysis (Table 2). At multivariate analysis, a higher CD4^+^ % was associated with a higher probability to obtain inhibition (adjusted odd ratio 1.12, 95% CI 1.001–1.254, *P* = 0.047), after adjusting for absolute CD4^+^ count and CD4/CD8 ratio.

**Table 2:** Variables associated with the percentage of inhibition positive PLWHIV at 2 months post boost dose (+60 v2) (univariate and multivariate logistic regression analysis).

	Univariate analysis	*P*	Multivariate analysis	*P*
	OR (95% CI)		Adjusted OR (95% CI)	
Age, per +10 years	1.36 (0.67–2.79)	0.395		
Male gender	1.00 (0.18*–*5.70)	1.000		
Type of vaccine (mRNA-1273 vs. BNT162b2)	1.43 (0.30–6.88)	0.655		
BMI, kg/m^2^	1.07 (0.84–1.37)	0.577		
IDU	0.90 (0.09–8.89)	0.926		
Years from HIV infection, per +10 years	1.09 (0.52–2.26)	0.819		
CDC stage C	0.32 (0.06–1.63)	0.169		
HBV or HCV coinfection	2.06 (0.23–18.78)	0.522		
Zenith HIV-RNA, per +1 log copies/mL	0.46 (0.10–2.10)	0.315		
CD4 cell count at nadir, per +100 cell/mmc	1.20 (0.74–1.93)	0.450		
Years from first ART, per +10 years	1.73 (0.46–6.51)	0.418		
Type of ART:				
-InSTI + 2NRTI	Ref			
-PI + 2NRTI	1.56 (0.15–16.72)	0.712		
-NNRTI + 2NRTI	1.40 (0.23–8.78)	0.715		
Baseline HIV-RNA < 50 cp/mL	1.95 (0.18–21.54)	0.585		
Time from last HIV-RNA > 50 copies/mL, per +10 years	4.27 (0.24–76.54)	0.325		
CD4 cell count at baseline, per +100 cell/mmc	0.85 (0.66–1.09)	0.193	0.89 (0.67–1.17)	0.389
CD4 cell count at baseline <= 350 cell/mmc	0.26 (0.05–1.40)	0.117		
CD4%, per +1% increase	1.09 (0.01–1.18)	**0.029**	1.12 (1.001–1.254)	**0.047**
CD4% >= 30%	2.41 (0.51–11.37)	0.267		
CD4/CD8 ratio, per +1 increase	1.99 (0.45–8.85)	0.369	0.55 (0.14–2.13)	0.387
CD4/CD8 ratio >= 1	2.08 (0.38–11.48)	0.402		
Comorbidities:				
Diabetes	0.51 (0.05–5.65)	0.585		
Hypertension	2.06 (0.23–18.78)	0.522		
Chronic lung disease	nc	0.999		
Previous cancer	1.11 (0.12–10.65)	0.931		

Bold values indicate statistical significance *p* ≤ 0.05.

*ART* antiretroviral therapy, *BMI* body mass index, *CI* confidence intervals, *HBV* hepatitis B virus, *HCV* hepatitis C virus, *IDU* injecting drug users, *InSTI* integrase strand transfer inhibitors, *MSM* men who have sex with men, *NRTI* nucleoside reverse transcriptase inhibitors, *NNRTI* non-nucleoside reverse transcriptase inhibitors, *OIR* optimal immunological recovery, *PI* protease inhibitors, *nc* not calculable.

### Spike-specific memory B cells following SARS-CoV-2 mRNA vaccination in PLWHIV

**Fig. 3:**
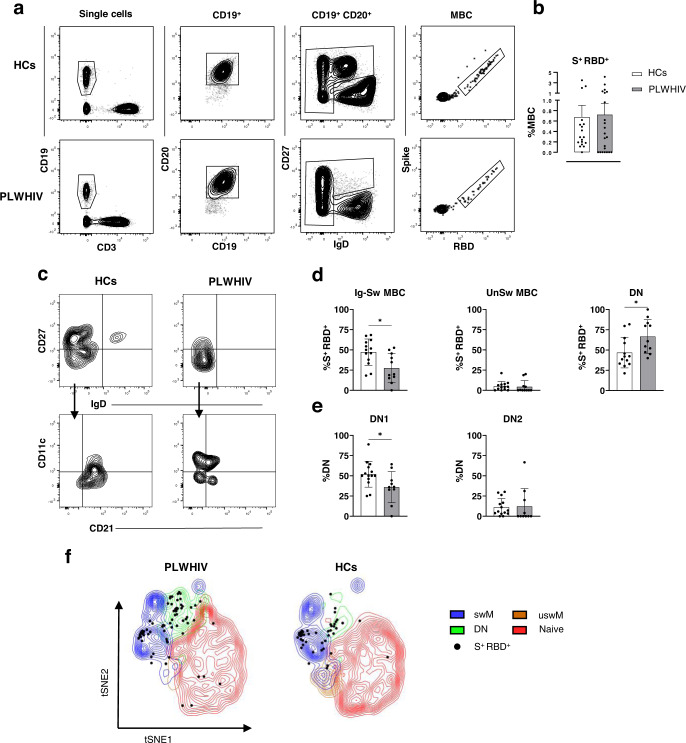
Analysis of spike-specific memory B cells at 6 months after the beginning of the vaccination cycle (+150 v2). **a** Gating strategy applied for identifying S^+ ^RBD^+^ MBC in PLWHIV and HCs. **b** Percentages of S^+^ RBD^+^ cells identified within MBC pool in each group. **c** Contour plot analysis of CD27 vs. IgD within S^+^ RBD^+^ MBC, for identifying Ig-switched (IgD^-^CD27^+^), Unswitched (IgD^+^CD27^+^) and double-negative (DN) (IgD^-^CD27^-^) MBC. The DN MBC were dissected according to CD11c and CD21 expression for the identification of DN1 (CD11c^-^CD21^+^) and DN2 (CD11c^+^CD21^-^) **d-e** Percentages of S^+^ RBD^+^ MBC subsets identified in c. Contour plot in a and c are representative from a single subject from each group. Statistical difference was assessed by Mann–Whitney test; *P ≤ 0.05. **f** t-SNE visualization of B cells and antigen-specific B cells in PLWHIV and HCs. t-SNE dimensionality reduction was used to visualize in a bidimensional space a multiparametric dataset. Major B-cell subsets (switched memory, swM; unswitched memory uswM; double negative, DN; and naive) from PLWHIV (left) and HCs (right) were displayed as contour plots in t-SNE map according to the expression of all analyzed markers. S^+ ^RBD^+^ B cells were shown as black dots. Sample size **b**, **f**: PLWHIV (*n*  =  22), HCs (*n*  =  18). Sample size **c**-**e**: PLWHIV (*n*  =  10), HCs (*n*  =  13). All samples were biologically independent.

The induction and persistence of spike-specific memory B cells (MBC), elicited by SARS-CoV-2 vaccination in PLWHIV was evaluated by multiparametric flow cytometry on PBMCs collected at +150 v2 (Fig. 3). Circulating spike-specific cells were identified as cells simultaneously positive for  PE-conjugated spike protein (S) and APC-conjugated RBD. Antigen-specific (S^+ ^RBD^+^) MBC were identified among CD19^+^ CD20^+^, after the exclusion of the IgD^+^ CD27^-^ naive population, applying the gating strategy reported in Fig. 3a. The mean frequency of S^+ ^RBD^+^ B cells observed in blood of PLWHIV at +150 v2 was not significantly different compared to HCs (0.7225 ± 1.032% vs. 0.6772 ± 0.9432%; *P* = 0.710) even though 36% of PLWHIV showed a very low amount of spike-specific B cells (< 0.1 % over all B cells) (Fig. 3b).

S^+^RBD^+^ B cells were dissected again to identify different populations of MBC according to IgD and CD27 expression (Fig. 3c). Spike-specific IgD^-^ CD27^+^ Ig-switched were recorded at a significant lower rate in PLWHIV compared to HCs (27.49 ± 17.81% vs. 47.27 ± 16.22%, respectively; *P* ≤ 0.05), suggesting a limited induction of this population or a reduced survival through time (Fig. 3d). On the contrary, the IgD^-^ CD27^-^ DN MBC, typically associated with an exhausted memory phenotype^31^, represented a significantly higher population in PLWHIV compared to HCs (66.72 ± 20.67% vs. 46.82 47.32 ± 18.85 %; *P* ≤ 0.05). Among DN subset, spike-specific DN1 cells were predominant compared to DN2 in both PLWHIV and HCs, even though the frequency of DN1 in PLWHIV was significantly lower than in HCs (35.98 ± 19.23% vs. 51.99 ± 16.07%; *P* ≤ 0.05; Fig. 3c, e).

The phenotypic distribution was confirmed by the t-SNE dimensionality reduction analysis (Fig. 3f), which showed a higher frequency of DN MBC in PLWHIV, while switched MBC were more abundant in HCs. A higher number of spike-specific B cells (dark spot) fell in the DN region of PLWHIV compared to healthy controls where the switched memory phenotype is predominant.

## Discussion

In the present study, we characterized the spike-specific antibody and memory B-cell responses in PLWHIV on ART vaccinated with two doses of a SARS-CoV-2 mRNA vaccine in the 6 months following vaccination and we compared them to HCs.

Almost all PLWHIV developed significant spike-specific plasma IgG titers after the first vaccine administration that significantly increased upon the second dose, remarking the immunogenicity of mRNA vaccines for these patients. Interestingly, anti-spike plasma IgG titers were still significant 6 months after the first dose compared to baseline in most patients. Differently from what observed by other authors^22,32,33^, we did not record any statistical difference in antigen-specific circulating IgG when comparing PLWHIV to the HCs at all time points. However, the strength of the antibody response could be affected by the different vaccine formulations used, age and gender^8,34^. Indeed, in the study by Portillo et al. the group of healthy volunteers was composed by very young subjects (median age 30 years vs. 54 years of PLWHIV) that could develop higher immune responses^22^. Xu and colleagues, as part of a larger clinical study, reported the IgG measured 2 weeks after second vaccine dose while assessing humoral response at longer time points (e.g., 60 days from second dose) might have found comparable responses between cohorts^32^. Finally, the preliminary study of Jedicke et al. characterized the response at only two non-standardized time points (after priming and after boost) and, as declared by the authors, suffered for non-perfect demographic match between cohorts^33^. On the other hand, our findings are consistent with those by Antinori et al. and Lombardi et al.^13,35^, despite again in these studies the HCs and PLWHIV age were un-matched and antibody responses were evaluated only up to 1 month after the second vaccine dose. Finally, the study by Tau et al.^36^ did not show differences between HCs and PLWHIV, but the antibody response in PLWHIV was measured at a time point that was closer to the second vaccine dose, compared to HCs. In general, when compared to data available in current literature, the strength of our work is the inclusion of a HCs group well balanced with PLWHIV in terms of age, by the length of follow-up and by the standardization of sampling time during follow-up.

The CD4^+^ T cell count was shown to inversely correlate with  enhanced morbidity and mortality for COVID-19 in PLWHIV ^37^. We observed that the anti-SARS-CoV-2 IgG response of subjects in the Low CD4^+^ T cell (≤ 350/mmc) subgroup was generally low, in line to what reported previously^14^, possibly reflecting a reduced ability to develop a strong antibody response after the boost. Lombardi and colleagues adopted a similar stratification according to CD4^+^  T cell count^35^. In contrast to them, we did find a reduced antibody production at +30 v2 in the Low CD4^+^ T cell subgroup. It is important to note that the limited number of subjects within CD4^+^ T cell subgroups precludes further considerations, and this result should be corroborated by larger studies. We also found a significant positive correlation, according to Spearman test, between CD4^+^ T cells/mmc and antibody titers at pre v2 and +30 v2.

Within antigen-specific antibodies, a pivotal role is played by neutralizing immunoglobulins. Previous work measured neutralization capacity within 1 month after the second dose finding either no differences compared to HCs^35,38^ or reduced neutralization in PLWHIV with lower CD4^+^ count/mmc compared to the others^13^. Jedicke and colleagues, though with non-standardized sampling schedule, associated the CD4/CD8 Tcell ratio ≤ 0.5 to reduced neutralizing antibodies after the second mRNA vaccine dose^33^. In our study, we assessed the ACE2/RBD binding inhibition activity after the second dose at two distant time points to characterize its persistence. We found that more than 80% of PLWHIV showed a positive ACE2/RBD binding inhibition activity at +60 v2, that declined up to 54% at +150 v2. This reduction observed in PLWHIV can reflect their chronic immune impairment and may be a hint of reduced immune persistence. Differently from what previously reported by Antinori and colleagues^13^, when PLWHIV were stratified according to CD4^+^ T cell count no significant differences were observed for mean ACE2/RBD binding inhibition percentages nor for percentage of neutralization-positive samples. However, the percentage of inhibition positive PLWHIV seemed to be correlated to the CD4^+^ T cells percentage.

While SARS-CoV-2-specific antibody response has long been considered the principal immune component in the context of COVID-19 vaccination, many studies have now recognized the crucial role of the B and T responses, especially for the induction and persistence of immune memory^39–41^. Recent studies have demonstrated the presence of spike-specific T cells in PLWHIV vaccinated with two doses of mRNA vaccine, with no differences for most PLWHIV compared to HCs^13,36,38^. Here, we demonstrated that spike-specific B cells were at a comparable rate in PLWHIV and HCs, but with different phenotypes. IgD^- ^CD27^+^ Ig-switched cells were recorded at a significant lower frequency in PLWHIV compared to HCs, as a result of a possible limited induction of this populations or a reduced survival through time, while IgD^-^ CD27^-^ DN cells were significantly higher. This alteration in the spike-specific MBC phenotypes reflects the atypical phenotypes observed in the B cell compartment of PLWHIV^42^. Even though CD27 is considered a marker expressed by antigen experienced B cells^43^, the DN population has been described as a dominant phenotype in many autoimmune disease^44^, chronic infection including HIV^24^, and elderly^31^. DN cells show signatures of antigen experienced B cells as shown by somatic hypermutation of their Ig genes, although at lower mutation load than classical switched memory B cells^45^. Two different phenotypes, named DN1 (CD11c^-^ CD21^+^) and DN2 (CD21^-^ CD11c^+^) have been described^46^. DN1 cells are the large majority of DN cells in healthy subjects^47^, where they may represent early activated memory cells, a subset that undergoes the germinal center (GC) affinity maturation step and eventually turns into Ig-Switched MBC^42^. On the contrary, the DN2 subset has been shown to be dominant in autoimmune diseases, and has been associated with exhausted or dysfunctional cells, due to the expression of inhibitory receptors of BCR signaling^48^. Accordingly, the DN2 subset likely derives from newly activated naïve cells that go through an extra-follicular differentiation pathway^42^. Here, among the spike-specific DN cells developed by PLWHIV, we observed a predominant CD21^+^ CD11^-^ DN1 phenotype, even though DN2 were detected, with a higher frequency compared to HCs.

The significant lower frequency of spike-specific Ig-switched MBC, which represent the larger subset in SARS-CoV-2 vaccinated subjects^17^, suggests a suboptimal response elicited by vaccination in PLWHIV. Indeed, Ig-switched MBCs are the product of expansion and clonal selection of antigen-specific B cells within the GC, in which the production of increasingly affine antibodies is optimized, whereas DN cells seem to undergo a reduced affinity maturation process. We can therefore speculate that the functionality of the antibodies produced by PLWHIV can be different from what observed in HCs, due to the defective B cell memory compartment. This could explain the different behavior observed in Ab binding inhibition activity at day +150 v2, by which time many PLWHIV lost the ability to inhibit RBD/ACE2 binding.

As this is the first time that spike-specific memory B cells are evaluated in PLWHIV after mRNA COVID-19 vaccine, the role of impaired B memory is not known yet and deserves to be deepened with further studies. Findings on other immunocompromised subjects suggest that a third dose can induce a strong immune response even in patients who did not develop immunity after the second dose^9,49,50^ and a recent work on PLWHIV assessed a general strong humoral response to the third dose but with a reduced T-cell stimulation compared to HCs^51^. New investigations are required to elucidate the effect of multiple vaccine boosting in PLWHIV.

This study has some limitations. The enrollment process, samples collection and analysis were not blinded since the cohorts of PLWHIV and HCs are part of two different clinical studies. Volunteers from the PLWHIV cohort were recruited only among patients treated within the Infectious and Tropical Diseases Unit, Azienda Ospedaliera Universitaria Senese (Siena, Italy). Consequently, we can not exclude this may have biased our enrolling procedure. Even though it was not possible to match PLWHIV and HCs for gender ratio and BMI, when we analyzed correlates of immunological endpoints in PLWHIV, gender and BMI did not show an association with any outcome. The majority of HCs were vaccinated with BNT162b2 vaccine while PLWHIV mostly received mRNA-1273 and differences in mRNA content may have affected the strength of immune response^34^. Since the difficulties in enrolling volunteers within PLWHIV cohort and a decreased adherence to the sampling schedule, sample size was lower than expected. For this reason, very small differences observed between cohorts were not marked as significant.

Owing to the small number of subjects in the Low and Average CD4^+^ subgroups, we could only make speculations on reduced ability in mounting a humoral immune response. Our cohort represents the general condition of PLWHIV in high-income countries, nevertheless a larger study on people with Low CD4^+^ T cells, uncontrolled HIV and AIDS would be beneficial to describe a more complete scenario.

In summary, our study provides real-world data on immunogenicity of SARS-CoV-2 mRNA vaccines in PLWHIV up to 6 months after vaccination. The data show that the spike-specific antibody response of PLWHIV is comparable, in quantitative terms, to that induced by vaccination in HCs. However, HIV patients showed a higher frequency of spike-specific IgD^-^ CD27^-^ DN and a significant lower rate of IgD^-^ CD27^+^ Ig-switched compared to HCs. These aspects suggest a different maturation of the B response in the GC with reduced functionality of the antigen-specific memory B cells and deserve to be explored for future research. These data remark the importance of tailored vaccination policies for PLWHIV.

## Supplementary information

Supplementary Information

Supplementary Data 1

Description of Additional Supplementary Files

Reporting Summary

## Data Availability

Data that underlie the graphs and results reported in this article are available in Supplementary Data 1. Individual participant clinical data are available, after de-identification, upon reasonable request.
