# Supplementary material for: B cell response after SARS-CoV-2 mRNA vaccination in people living with HIV
Source: Commun Med (Lond). 2023 Jan 30;3:13. doi: 10.1038/s43856-023-00245-5 (PMC9886211; doi:10.1038/s43856-023-00245-5)
Supplement: Supplementary file 1 — Supplementary Information [file 43856_2023_245_MOESM1_ESM.pdf]

Supplementary Material

B cell response after SARS-CoV-2 mRNA vaccination in people living with HIV

Jacopo Polvere [1], Massimiliano Fabbiani [2], Gabiria Pastore [1], Ilaria Rancan [2,3], Barbara Rossetti [2], Miriam Durante [3], Sara Zirpoli [1], Enrico Morelli [3], Elena Pettini [1], Simone Lucchesi [1], Fabio Fiorino [1], Mario Tumbarello [2,3], Annalisa Ciabattini [1], Francesca Montagnani [2,3]* and Donata Medaglini [1]*

1 Laboratory of Molecular Microbiology and Biotechnology, Department of Medical Biotechnologies, University of Siena; Siena, Italy.

2 Department of Medical Sciences, Infectious and Tropical Diseases Unit, University Hospital of Siena; Siena, Italy.

3 Department of Medical Biotechnologies, University of Siena; Siena, Italy.

* Correspondence:

Donata Medaglini: donata.medaglini@unisi.it, +39 0577 233307

Francesca Montagnani: francesca.montagnani@unisi.it, +39 0577 586562

Keywords: HIV, vaccination, SARS-CoV-2, COVID-19, memory B cells.

Table of contents

Supplementary table 1. Baseline characteristics of people living with HIV (PLWHIV) and healthy controls (HCs). 2

Supplementary table 2. Variables associated to ACE2/RBD inhibition percentage at 2 months post second dose (+60 v2) in PLWHIV (univariate and multivariate linear regression analysis). 3

Supplementary figure 1. Spike-specific antibody response stratified according to $CD4^+$/mmc cells count. **S**tatistical differences between groups at each time point. 4

Supplementary table 1. Baseline characteristics of people living with HIV (PLWHIV) and healthy controls (HCs).

	PLWH<br>N=84	HC<br>N=79	P
**General variables**			
Age, years	52 (46-58)	52 (45-60)	0.732
Male gender	64 (76.2)	22 (27.8)	**<0.001**
Type of vaccine:			
-mRNA-1273	43 (51.2)	10 (12.7)	
-BNT162b2	41 (48.8)	69 (87.3)	**<0.001**
BMI, Kg/m^2	25.1 (22.9-29.7)	23.7 (20.8-26.8)	**0.037**
**HIV-related variables**			
Risk factor for HIV infection:			
-Heterosexual	14 (16.7)	-	
-MSM	34 (40.5)	-	
-IDU	10 (11.9)	-	
-Other/unknown	26 (31.0)	-	
Years from HIV infection	10.5 (6.3-24.8)	-	
CDC stage C	16 (19.0)	-	
HBV or HCV coinfection	21 (25.0)	-	
Zenith HIV-RNA, log10 copies/mL	5.22 (4.66-5.69)	-	
CD4 cell count at nadir, cell/mmc	154 (34-302)	-	
Years from first ART	7.9 (5.3-13.9)	-	
Type of ART:			
-InSTI + 2NRTI	36 (42.9)	-	
-PI + 2NRTI	6 (7.1)	-	
-NNRTI + 2NRTI	20 (23.8)	-	
-Other	22 (26.2)	-	
Baseline HIV-RNA <50 copies/mL	76 (90.5)	-	
Time from last HIV-RNA >50 copies/mL, years	4.9 (1.6-8.0)	-	
CD4 cell count at baseline, cell/mmc	639 (425-842)	-	
- <=350 cell/mmc	14 (16.7)	-	
- 350-500 cell/mmc	13 (15.5)	-	
- >=500 cell/mmc	57 (67.9)	-	
CD4%	30.8 (23.7-37.8)	-	
CD4%>=30%	47 (56.0)	-	
CD4/CD8 ratio	0.8 (0.53-1.10)	-	
CD4/CD8 ratio >=1	31 (36.9)	-	
OIR	28 (33.3)	-	
Comorbidities in HIV-infected patients:			
Diabetes	5 (6.0)	-	
Hypertension	14 (16.7)	-	
Chronic lung disease	7 (8.3)	-	
Previous cancer	7 (8.3)	-	

Notes: values are expressed as n (%), except for * median (interquartile range)

Abbreviations: ART, antiretroviral therapy; BMI, body mass index; HBV, hepatitis B virus; HCV, hepatitis C virus; IDU, injecting drug users; InSTI, integrase strand transfer inhibitors; MSM, men who have sex with men; NRTI, nucleoside reverse transcriptase inhibitors; NNRTI, non nucleoside reverse transcriptase inhibitors; OIR, optimal immunological recovery; PI, protease inhibitors.

Supplementary table 2. Variables associated to ACE2/RBD inhibition percentage at 2 months post second dose (+60 v2) in PLWHIV (univariate and multivariate linear regression analysis).

	**Univariate analysis**		**Multivariate analysis**	
	**Mean change (95% CI)**	**P**	**Adjusted mean change (95% CI)**	**P**
Age, per +10 years	5.01 (-2.85 / 13.03)	0.204		
Male gender	-6.18 (-25.65 / 13.29)	0.527		
Type of vaccine (mRNA-1273 vs BNT162b2)	0.06 (-18.28 / 18.39)	0.995		
BMI, Kg/m^2	0.47 (-1.06 / 2.00)	0.535		
IDU	-1.46 (-27.95 / 25.03)	0.912		
Years from HIV infection, per +10 years	0.76 (-7.56 / 9.08)	0.855		
CDC stage C	-16.45 (-37.41 / 4.51)	0.121		
HBV or HCV coinfection	3.21 (-17.50 / 23.91)	0.757		
Zenith HIV-RNA, per +1 log copies/mL	-2.09 (-17.91 / 13.73)	0.789		
CD4 cell count at nadir, per +100 cell/mmc	0.99 (-4.52 / 6.50)	0.718		
Years from first ART, per +10 years	6.98 (-4.35 / 18.31)	0.221		
InSTI + 2NRTI	-6.08 (-23.24 / 11.09)	0.480		
Baseline HIV-RNA <50 cp/mL	14.94 (-16.54 / 46.42)	0.345		
Time from last HIV-RNA >50 copies/mL, per +10 years	20.24 (-6.55 / 47.04)	0.134		
CD4 cell count at baseline, per +100 cell/mmc	-0.05 (-2.83 / 2.72)	0.969		
CD4 cell count at baseline <=350 cell/mmc	-19.30 (-40.99 / 2.40)	0.080	-17.18 (-41.54 / 7.18)	0.163
CD4%>=30%	8.08 (-8.81 / 24.97)	0.341	-2.18 (-26.54 / 22.18)	0.858
CD4/CD8 ratio >=1	10.34 (-6.81 / 27.49)	0.232	7.07 (-17.24 / 31.39)	0.561

Abbreviations: ART, antiretroviral therapy; BMI, body mass index; CI, confidence intervals; HBV, hepatitis B virus; HCV, hepatitis C virus; IDU, injecting drug users; InSTI, integrase strand transfer inhibitors; MSM, men who have sex with men; NRTI, nucleoside reverse transcriptase inhibitors; NNRTI, non nucleoside reverse transcriptase inhibitors; OIR, optimal immunological recovery; PI, protease inhibitors.

Supplementary figure 1. Spike-specific antibody response stratified according to CD4+/mmc cells count.

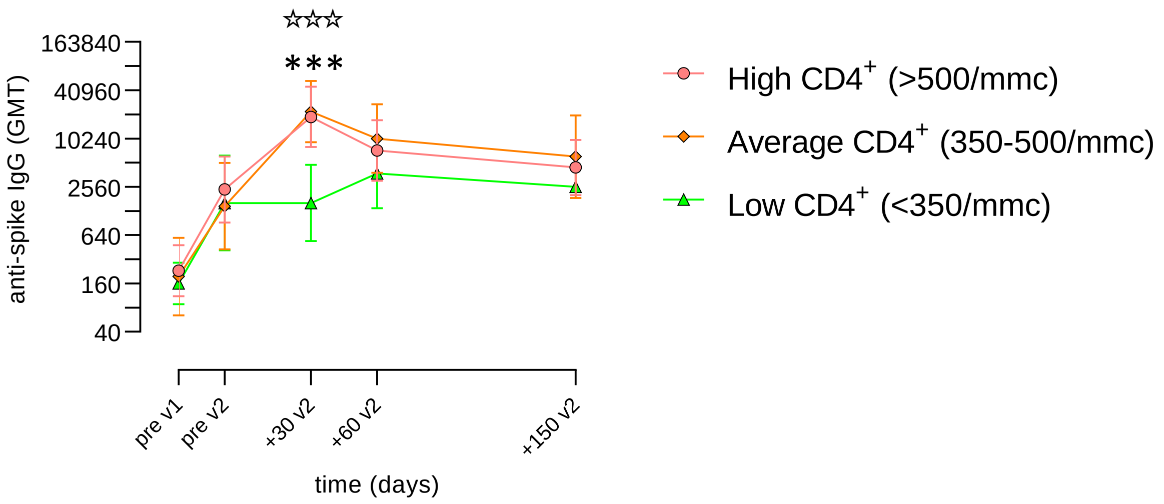

Antibody titres are expressed as the reciprocal of the dilution of sample reporting a double OD value compared to the background. Data are presented as geometric mean titers (GMT) with 95% CI. **S**tatistical differences between groups at each time point were assessed with Mann-Whitney test. ***P ≤ 0.001 (Average CD4+ vs Low CD4+); ☆☆☆P ≤ 0.001 (High CD4+ vs Low CD4+). Sample size High CD4+: pre v1 (n = 36), pre v2 (n = 27), +30 v2 (n = 38), +60 v2 (n = 51), +150 v2 (n = 15). Sample size Average CD4+: pre v1 (n = 7), pre v2 (n = 5), +30 v2 (n = 9), +60 v2 (n = 6), +150 v2 (n = 4). Sample size Low CD4+: pre v1 (n = 9), pre v2 (n = 6), +30 v2 (n = 6), +60 v2 (n = 9), +150 v2 (n = 3). All samples were biologically independent.
