# Supplementary material for: B cell response after SARS-CoV-2 mRNA vaccination in people living with HIV
Source: Commun Med (Lond). 2023 Jan 30;3:13. doi: 10.1038/s43856-023-00245-5 (PMC9886211; doi:10.1038/s43856-023-00245-5)
Supplement: Supplementary file 3 — Description of Additional Supplementary Files [file 43856_2023_245_MOESM3_ESM.pdf]

Description of Additional Supplementary Files

File Name: Supplementary Data 1

Description: Data for figure 2 and 3 and for Supplementary figure 1.
