# Supplementary material for: B cell response after SARS-CoV-2 mRNA vaccination in people living with HIV
Source: Commun Med (Lond). 2023 Jan 30;3:13. doi: 10.1038/s43856-023-00245-5 (PMC9886211; doi:10.1038/s43856-023-00245-5)
Supplement: Supplementary file 4 — Reporting Summary [file 43856_2023_245_MOESM4_ESM.pdf]

nature research

Corresponding author(s): Donata Medaglini

Last updated by author(s): Nov 18, 2022

Reporting Summary

Nature Research wishes to improve the reproducibility of the work that we publish. This form provides structure for consistency and transparency in reporting. For further information on Nature Research policies, see our Editorial Policies and the Editorial Policy Checklist.

Statistics

For all statistical analyses, confirm that the following items are present in the figure legend, table legend, main text, or Methods section.

n/a	Confirmed	
☐	☒	The exact sample size (*n*) for each experimental group/condition, given as a discrete number and unit of measurement
☐	☒	A statement on whether measurements were taken from distinct samples or whether the same sample was measured repeatedly
☐	☒	The statistical test(s) used AND whether they are one- or two-sided<br>*Only common tests should be described solely by name; describe more complex techniques in the Methods section.*
☐	☒	A description of all covariates tested
☐	☒	A description of any assumptions or corrections, such as tests of normality and adjustment for multiple comparisons
☐	☒	A full description of the statistical parameters including central tendency (e.g. means) or other basic estimates (e.g. regression coefficient) AND variation (e.g. standard deviation) or associated estimates of uncertainty (e.g. confidence intervals)
☐	☒	For null hypothesis testing, the test statistic (e.g. *F*, *t*, *r*) with confidence intervals, effect sizes, degrees of freedom and *P* value noted<br>*Give P values as exact values whenever suitable.*
☒	☐	For Bayesian analysis, information on the choice of priors and Markov chain Monte Carlo settings
☒	☐	For hierarchical and complex designs, identification of the appropriate level for tests and full reporting of outcomes
☐	☒	Estimates of effect sizes (e.g. Cohen's *d*, Pearson's *r*), indicating how they were calculated

Our web collection on statistics for biologists contains articles on many of the points above.

Software and code

Policy information about availability of computer code

Data collection	REDCap (Research Electronic Data Capture, Vanderbilt University, TN, USA)
Data analysis	GraphPad Prism v9 (GraphPad Software, San Diego, CA, USA)<br>SPSS Software, version 23.0 (SPSS Inc., Chicago, IL, USA)<br>Rtsne package 0.15, R software (R Foundation for Statistical Computing, Vienna, Austria)

For manuscripts utilizing custom algorithms or software that are central to the research but not yet described in published literature, software must be made available to editors and reviewers. We strongly encourage code deposition in a community repository (e.g. GitHub). See the Nature Research guidelines for submitting code & software for further information.

Data

Policy information about availability of data

All manuscripts must include a data availability statement. This statement should provide the following information, where applicable:

- Accession codes, unique identifiers, or web links for publicly available datasets
- A list of figures that have associated raw data
- A description of any restrictions on data availability

Data underlie graphs reported in this work are available in Supplementary Data 1. Individual participant clinical data that underlie the results reported in this article, after de-identification, are available upon request.

Field-specific reporting

Please select the one below that is the best fit for your research. If you are not sure, read the appropriate sections before making your selection.

☒ Life sciences ☐ Behavioural & social sciences ☐ Ecological, evolutionary & environmental sciences

For a reference copy of the document with all sections, see nature.com/documents/nr-reporting-summary-flat.pdf

Life sciences study design

All studies must disclose on these points even when the disclosure is negative.

Sample size	84 people living with HIV (PLWHIV) and 79 healthy donors were enrolled in the study. The sample size was not determined in advance but consist in PLWHIV treated by the Infectious and Tropical Diseases Unit of Le Scotte hospital in Siena that accepted to participate as volunteers. However, they represent the majority of PLWHIV treated by the hospital unit. A group of healthy donors with comparable numerosity was enrolled. The reduced sample size of our cohort was a limitation of this study
Data exclusions	Exclusion criteria were pregnancy, withdrawal of consent or refusal to participate (for both studies), administration of high-dose steroids or other immunosuppressant drugs, comorbidities associated with relevant immunosuppression (e.g. active cancer, organ transplantation) other than HIV itself, clinical problems for collecting additional blood samples beyond the amount required for routine care and participation to other clinical trials (for PATOVAC study), being affected by any immunocompromising condition (congenital, acquired, or drug-related; for IMMUNO_COV study)
Replication	All wet lab analysis were conducted in duplicate, except for flow cytometry due to limited sample availability. Assays were done throughout the entire time of the study in the same conditions (reagents, temperatures, machineries, protocols). Results were reproducible in different days and by different operators.
Randomization	No randomization was applied since samples have been preliminary classified according to HIV positivity status
Blinding	Due to logistic necessities it was not possible to apply blinding at the time of samples collection and samples analysis

Reporting for specific materials, systems and methods

We require information from authors about some types of materials, experimental systems and methods used in many studies. Here, indicate whether each material, system or method listed is relevant to your study. If you are not sure if a list item applies to your research, read the appropriate section before selecting a response.

Materials & experimental systems

n/a	Involved in the study	
☐	☒	Antibodies
☒	☐	Eukaryotic cell lines
☒	☐	Palaeontology and archaeology
☒	☐	Animals and other organisms
☐	☒	Human research participants
☐	☒	Clinical data
☒	☐	Dual use research of concern

Methods

n/a	Involved in the study	
☒	☐	ChIP-seq
☐	☒	Flow cytometry
☒	☐	MRI-based neuroimaging

Antibodies

Antibodies used	ELISA antibody: Anti-human horseradish peroxidase (HRP)-conjugated IgG (Southern Biotech)<br>Flow cytometry antibodies: CD3-BV650 (clone OKT3); CD21-FITC (clone B-LY4), CD19-BUV395 (clone SJ25C1), CD10-PECF594 (clone HI10A), IgM-BV605 (clone G20-127), IgD-BV711 (clone IA6-2), CD27-BV786 (clone O323), CD11c-BB700 (clone 3.9), CD20-APCH7 (clone 2H7), CD38-BUV737 (clone HB7), IgG-PE-Cy7 (clone G18-145, all from Becton Dickinson), IgA-Vio blue (clone IS11-8E10, Miltenyi Biotec).
Validation	Antibody for ELISA has been validate for use in ELISA as reported on the manufacturer website: https://www.southernbiotech.com/goat-anti-human-igg-hrp-2040-05<br>Flow cytometry antibodies has been validated by manufacturers, specifically Miltenyi Biotech (https://www.miltenyibiotec.com/IT-en/) and BD biosciences (https://www.bdbiosciences.com/en-us).<br>Antibodies profiles are the following:<br>- CD3-BV650 (clone OKT3): https://www.bdbiosciences.com/en-ca/products/reagents/flow-cytometry-reagents/research-reagents/single-color-antibodies-ruo/bv650-mouse-anti-human-cd3.750984

-CD21-FITC (clone B-LY4): https://www.bdbiosciences.com/en-ca/products/reagents/flow-cytometry-reagents/research-reagents/single-color-antibodies-ruo/fitc-mouse-anti-human-cd21.561372
-CD19-BUV395 (clone SJ25C1): https://www.bdbiosciences.com/en-ca/products/reagents/flow-cytometry-reagents/research-reagents/single-color-antibodies-ruo/buv395-mouse-anti-human-cd19.563549
-CD10-PECF594 (clone HI10A): https://www.bdbiosciences.com/en-it/products/reagents/flow-cytometry-reagents/research-reagents/single-color-antibodies-ruo/pe-cf594-mouse-anti-human-cd10.562396
-IgM-BV605 (clone G20-127): https://www.bdbiosciences.com/en-it/products/reagents/flow-cytometry-reagents/research-reagents/single-color-antibodies-ruo/bv605-mouse-anti-human-igm.562977
-IgD-BV711 (clone IA6-2): https://www.bdbiosciences.com/en-it/products/reagents/flow-cytometry-reagents/research-reagents/single-color-antibodies-ruo/bv711-mouse-anti-human-igd.740794
-CD27-BV786 (clone O323): https://www.bdbiosciences.com/en-it/products/reagents/flow-cytometry-reagents/research-reagents/single-color-antibodies-ruo/bv786-mouse-anti-human-cd27.751676
-CD11c-BB700 (clone 3.9): https://www.bdbiosciences.com/en-it/products/reagents/flow-cytometry-reagents/research-reagents/single-color-antibodies-ruo/bb700-mouse-anti-human-cd11c.748270
-CD20-APCH7 (clone 2H7): https://www.bdbiosciences.com/en-it/products/reagents/flow-cytometry-reagents/research-reagents/single-color-antibodies-ruo/apc-h7-mouse-anti-human-cd20.560734
-CD38-BUV737 (clone HB7): https://www.bdbiosciences.com/en-it/products/reagents/flow-cytometry-reagents/research-reagents/single-color-antibodies-ruo/buv737-mouse-anti-human-cd38.612824
-IgG-PE-Cy7 (clone G18-145): https://www.bdbiosciences.com/en-it/products/reagents/flow-cytometry-reagents/research-reagents/single-color-antibodies-ruo/pe-cy-7-mouse-anti-human-igg.561298
-IgA-Vio blue (clone IS11-8E10): https://www.miltenyibiotec.com/IT-en/products/iga-antibody-anti-human-is11-8e10.html#pure:100-ug-in-1-ml

Human research participants

Policy information about studies involving human research participants

Population characteristics

A total of 84 PLWHIV with a median age of 52 years (IQR 46-58) were enrolled in the study, of whom 64 (76.2%) were male and 20 (23.8%) were female. The control group (HCs) was composed of 79 healthy volunteers with a median age of 52 years (IQR 45-60) of whom 22 (27.8%) were males and 57 (72.2%) were females. A slightly higher body mass index (BMI) (25.1 vs 23.7, p=0.037) was observed in PLWHIV. HCs were more frequently vaccinated with BNT162b2 vaccine when compared to PLWHIV who mostly received mRNA-1273 (87.3% vs 48.8%, p<0.001.)

Recruitment

Study participants were recruited at the Infectious and Tropical Diseases Unit, Azienda Ospedaliera Universitaria Senese (Siena, Italy). All participants provided written informed consent before participation to the study. No self-selection bias nor other biases have been observed

Ethics oversight

Local Ethical Committee for Clinical Experimentation of Regione Toscana Area Vasta Sud Est (CEASVE)

Note that full information on the approval of the study protocol must also be provided in the manuscript.

Clinical data

Policy information about clinical studies
All manuscripts should comply with the ICMJE guidelines for publication of clinical research and a completed CONSORT checklist must be included with all submissions.

Clinical trial registration

Protocol code 19479 PATOVAC v1.0 of 03 Mar 2021, approved on 15 Mar 2021 and
Protocol code 18869 IMMUNO_COV v1.0 of 18 Nov 2020, approved on 21 Dec 2020

Study protocol

The study protocol is available upon request to the corresponding author (donata.medaglini@unisi.it)

Data collection

Setting of volunteer recruiting, data and sample collection: Infectious and Tropical Diseases Unit, Azienda Ospedaliera Universitaria Senese (Siena, Italy), from 15 March 2021 to 14 March 2023
Setting of samples analysis and data analysis: Laboratory of Molecular Microbiology and Biotechnology (LAMMB), Department of Medical Biotechnologies, University of Siena (Siena, Italy), from 15 March 2021 to 14 March 2023

Outcomes

The primary outcome of the study is the characterization of the immune response elicited by SARS-CoV-2 vaccination and its persistence in healthy volunteers and patients affected by different pathologies (fragile subjects). The secondary outcomes are the integrated analysis of clinical and immunological data and the comparison between immune responses elicited in healthy donors and fragile subjects.

Flow Cytometry

Plots

Confirm that:

☒ The axis labels state the marker and fluorochrome used (e.g. CD4-FITC).

☒ The axis scales are clearly visible. Include numbers along axes only for bottom left plot of group (a 'group' is an analysis of identical markers).

☒ All plots are contour plots with outliers or pseudocolor plots.

☒ A numerical value for number of cells or percentage (with statistics) is provided.

Methodology

Sample preparation	Venous blood samples were collected in heparin-coated blood tubes (BD Vacutainer). PBMCs were isolated by density-gradient sedimentation, using Ficoll-Paque (Lymphoprep, Meda, Italy). Isolated PBMC were then cryopreserved in a cell recovery medium [10% DMSO (Thermo Fisher Scientific) and 90% heat inactivated fetal bovine serum (Sigma Aldrich)] and stored in liquid nitrogen until used.<br>Two million of PBMCs from each sample were incubated with BD human FC block (BD Biosciences) and stained with biotinylated spike S1+S2 ECD-His recombinant biotinylated-protein (Sino Biological) conjugated with SA-R-Phycoerythrin (PE) and RBD recombinant biotinylated-protein (BioLegend) conjugated with SA-Allophycocyanin (APC), together with the following fluorescent antibodies: CD3-BV650 (clone OKT3); CD21-FITC (clone B-LY4), CD19-BUV395 (clone SJ25C1), CD10-PECF594 (clone HI10A), IgM-BV605 (clone G20-127), IgD-BV711 (clone IA6-2), CD27-BV786 (clone O323), CD11c-BB700 (clone 3.9), CD20-APCH7 (clone 2H7), CD38-BUV737 (clone HB7), IgG-PE-Cy7 (clone G18-145, all from Becton Dickinson), IgA-Vio blue (clone IS11-8E10, Miltenyi Biotec). All antibodies were titrated for optimal dilution. Following surface staining, cells were washed once with PBS and labeled with Zombie Aqua Fixable Viability Kit (Thermofisher) according to the manufacturer instruction. Cells were fixed in BD fixation solution (BD Biosciences) and acquired with SO LSRFortessa X20 flow cytometer (BD Biosciences). Data analysis was performed using FlowJo v10 (TreeStar, USA).
Instrument	SO LSRFortessa X20 flow cytometer (BD Biosciences)
Software	Data collection was done with BD FACSDiva (BD Biosciences, USA) and data analysis was conducted on FlowJo v10 (TreeStar, USA)
Cell population abundance	Approximately 2 x 10^6 PBMCs were stained and 0.7-1 x 10^6 cells were acquired and stored for each sample with SO LSRFortessa X20 flow cytometer. Since spike+ cells represent a rare population we evaluated only subjects with at least 10 cells detected in the respective antigen-specific (S+/RBD+) gate.
Gating strategy	PBMC were gated as live cells (LD- FSC-A), singlets (SSC-A FSC-A), CD19+ (CD19+ CD3-), B cells (CD19+CD20+), non-naive (IgD-CD27-, IgD- CD27+ and IgD+ CD27+), spike+ (S+ RBD+). Antigen -specific cells were gated according to CD27 and IgD espression to identify Ig-switched (IgD- CD27+), Ig-Unswitched (IgD+ CD27+) and double negative (IgD- CD27-) memory B cells. Double negative cells were gated again according to CD11c and CD21 expression, to identify double negative 1 CD11c-CD21+) and double negative 2 (CD11c+ CD21-) cells.

☒ Tick this box to confirm that a figure exemplifying the gating strategy is provided in the Supplementary Information.
